# 1371. Identification of Risk Factors to Predict *Gram negative bacteria* in Patients with Upper Extremity Infections

**DOI:** 10.1093/ofid/ofab466.1563

**Published:** 2021-12-04

**Authors:** Sophia Zhitomirsky, Hendrik Sy, Arsheena Yassin, Christine Stavropoulos, Andras Farkas

**Affiliations:** 1 Mount Sinai West Hospital, New York, New York; 2 Mount Sinai Morningside and West Hospitals, New York, New York; 3 Mount Sinai St. Luke’s Hospital, New York, NY; 4 Icahn School of Medicine at Mount Sinai St Luke’s and West Hospitals, New York, NY

## Abstract

**Background:**

Gram negative bacteria (GNB) have been identified as a cause of upper extremity infections and empiric treatment directed to both gram positive and negative organisms is often recommended. Risk-based approaches to establish need for gram-negative coverage may help to minimize unnecessary drug exposure, but further information on such methods are currently lacking. The aim of this study was to identify risk factors associated with the isolation of GNB in patients with upper extremity infections.

**Methods:**

We reviewed records of patients with upper extremity infections treated in two urban hospitals between March 2018 and July 2020. Prosthetic joint infections were excluded. Baseline demographic, clinical, surgical and microbiology data was collected. Multivariable logistic regression models were screened using Akaike Information Criterion to establish the best model and risk factors associated with isolation of a GNB.

**Results:**

We identified 111 patients, the majority of whom were male with frequent history of IV drug use. Deep wound cultures in 30 (33.3%) individuals yielded a GNB, and 80% of these cases were polymicrobial. Among the GNB, most prevalent were Enterobacterales (10.4%), HACEK group (6.39%), and *Pseudomonas spp.* (4.5%) (Tables 1. and 2.). Infections were mostly limited to the soft tissue structures of the hand and the forearm, with involvements of the joint and bone being second and third most common. The final model identified the use of IV medications (OR 4.14, 95% CI 1.3 - 14.46) together with prior surgery at the site of infection within the last year (OR 5.56, 95% CI 1.06 - 30.98), and having an open wound on presentation (OR 3.03, 95% CI 1.04 - 9.47) as factors independently associated with isolation of a GNB (Table 3). AUROC of 0.702 indicates acceptable model discrimination.

Table 1: Baseline characteristics

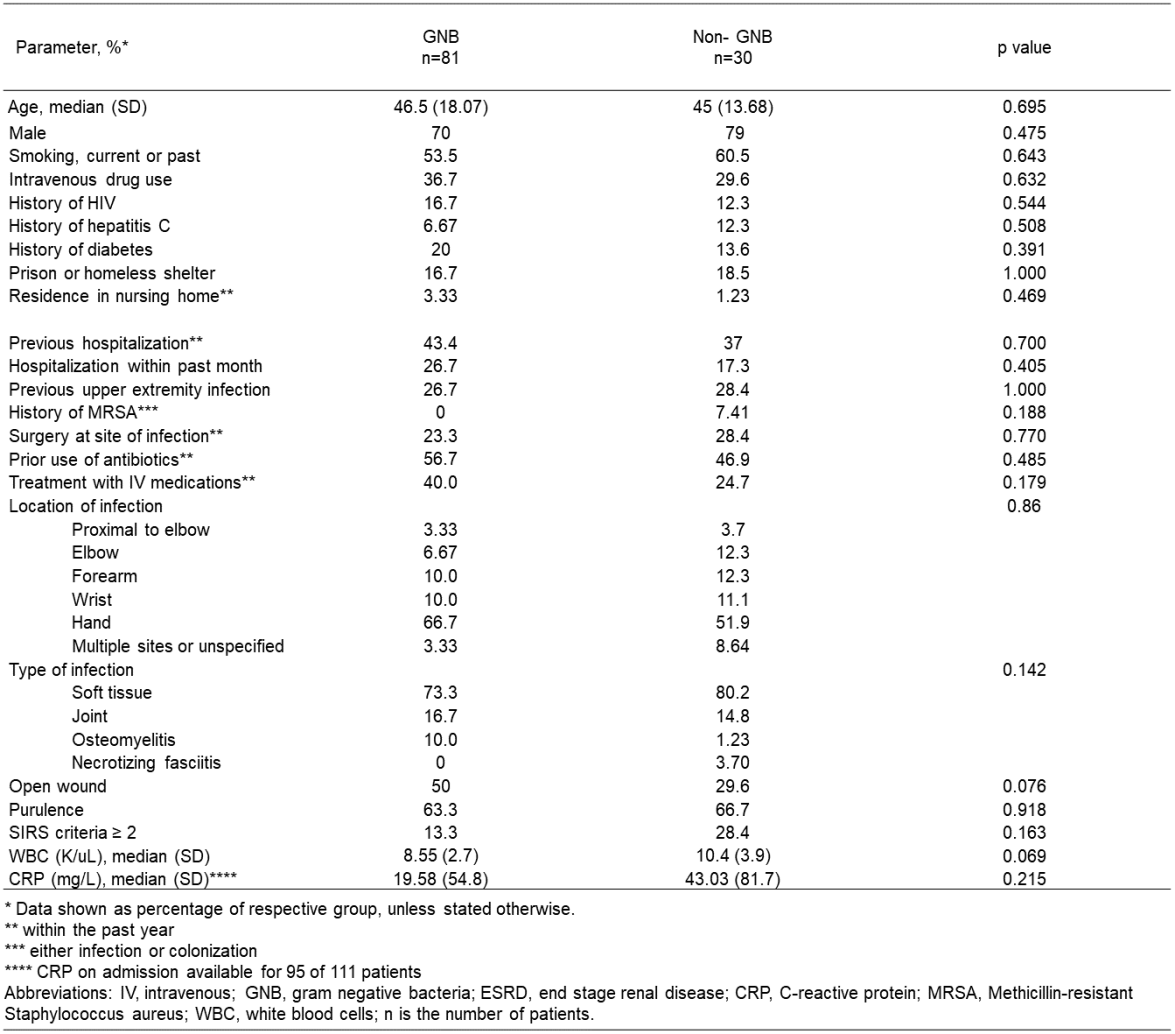

Table 2: Bacterial isolates

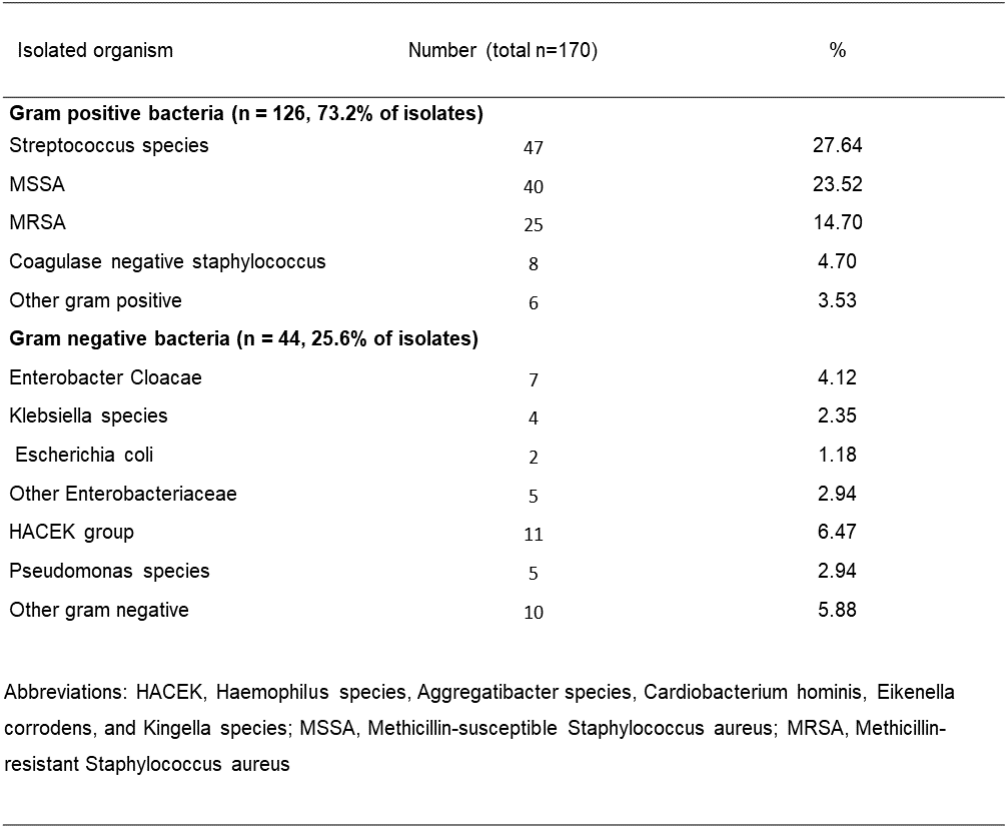

Table 3: Final model

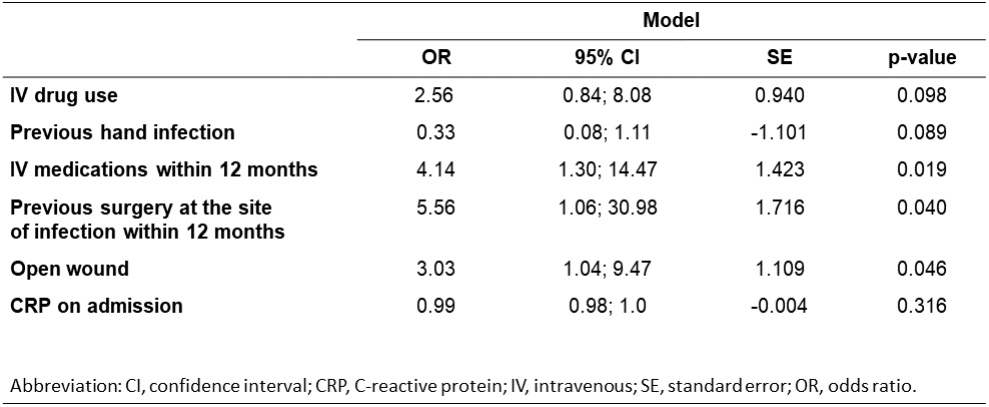

**Conclusion:**

Our logistic regression model identified significant predictors for isolation of GNB in upper extremity infections within this population. Results of this study will assist clinicians in making a better informed decision for the need of empiric gram negative coverage aimed to support the reduction of patient exposure to unnecessary antimicrobial coverage. External validation of the model is warranted prior to application to clinical care.

Figure 1: AUROC

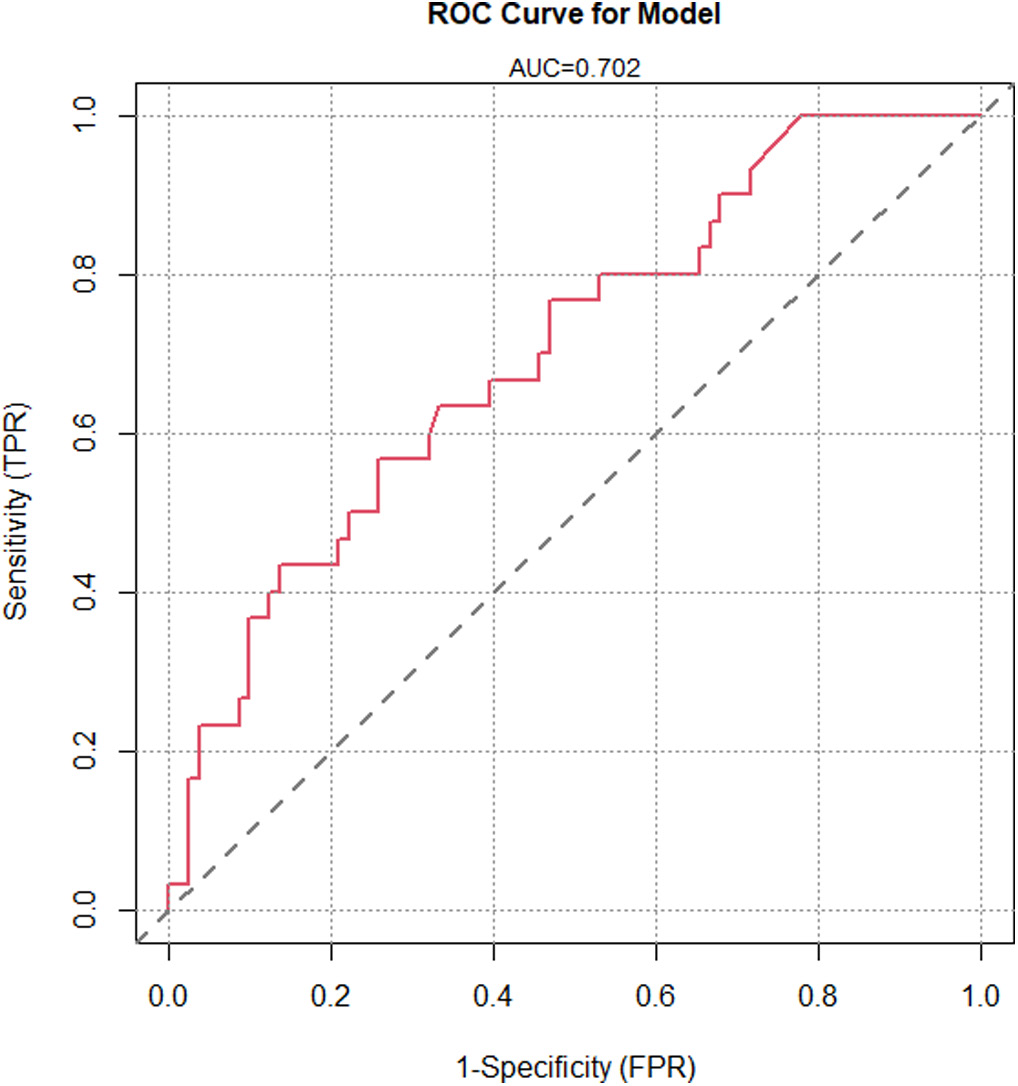

**Disclosures:**

**All Authors**: No reported disclosures

